# Correlation between lag screw route and the ideal insertion point of the intramedullary nail

**DOI:** 10.1038/s41598-021-93348-9

**Published:** 2021-07-02

**Authors:** Junya Yoshitani, Tamon Kabata, Yoshitomo Kajino, Daisuke Inoue, Takaaki Ohmori, Ken Ueoka, Yuki Yamamuro, Atsushi Taninaka, Hiroyuki Tsuchiya

**Affiliations:** Department of Orthopaedic Surgery, Kanazawa University Graduate School of Medical Sciences, 13-1 Takaramachi, Kanazawa, Ishikawa 920-8641 Japan

**Keywords:** Anatomy, Bone, Skeleton

## Abstract

Understanding the morphology of the superior aspect of the proximal femur is critical for treating femoral fracture. We assessed the correlation among the ideal insertion point of the femoral nail, femur head-neck axis, and native anteversion. One hundred patients with normal femurs were included in this study. Computed tomography (CT) images of the proximal femur superior aspect and amount of native anteversion were acquired. Generalised Procrustes analysis showed the morphological characteristics of the superior proximal femur according to native anteversion amount. Morphological characteristics were represented by 4 parameters; the correlation between parameters and native anteversion was investigated using CT data. The passing point of the line from the proximal femoral canal parallel to the native anteversion at the greater trochanter was located more posteriorly (mean 35.6%); the passing point of native anteversion was posterior in the femoral neck and head, although the line of the head-neck centre passed more anteriorly at the greater trochanter (mean 67.5%). This posterior translation was significantly associated with native anteversion amount. Morphometric geometric analysis showed that the lag screw could not pass head-neck centre from the nail inserted into proximal femoral canal. Anterior insertion of the nail was needed for positioning the lag screw centre.

Understanding the morphology of the superior aspect of the proximal femur is critical for safety and efficacy in orthopaedic surgery. For example, when inserting the short femoral nail to surgically treat intertrochanteric fractures of the femur or determining the entry point and anteversion of the femoral stem in total hip arthroplasty (THA), knowledge of the superior aspect of the proximal femur anatomy is necessary. Previous studies have investigated the morphology of the femur^1–6^. A cadaveric study investigated the greater trochanter morphology and assessed the optimal insertion point of the intramedullary nail; however, there was no information on a correlation between the route of the lag screw and the ideal insertion point of the intramedullary nail^5^. The route of the lag screw was influenced by femoral neck anteversion because it should be placed in the femoral neck and advanced to the femoral head centre. Therefore, we thought that the correlation between the entry point to the femoral canal and amount of femoral neck anteversion should be investigated as this could aid in correct insertion of the femoral nail. Furthermore, native femoral version can vary by as much as 60° among individuals^7^. There is a question on whether variation in amount of native anteversion influences the morphology of the proximal femur, thus affecting the entry point and route of the lag screw. In THA, determining the femoral version is important for the restoration of the native hip centre with optimisation of anteversion, which maximises stability and minimises impingement^8^. Intraoperatively, femoral anteversion is estimated by the angle between the stem neck and lower leg or determined by using a navigation system to provide the surgeons with accurate information on stem anteversion. However, there is no evidence that reproducing the native anteversion resulted in establishing a native hip centre. Therefore, this study investigated the correlation between the femoral head centre position and amount of native anteversion. We hypothesised a possible mismatch between the proximal femoral canal axis and femoral head-neck axis. Specifically, a mismatch exists between the intramedullary nail's ideal insertion point to the femoral canal and the central placement in the femoral neck of the lag screw. Further, the amount of native anteversion resulting in passing the femoral canal could not reproduce the native head centre. The purposes of this study were (1) to assess the morphology of the superior aspect of the proximal femur using morphometric geometric analysis, (2) to investigate the correlation between the true entry point to the femoral canal and amount of femoral native anteversion, and (3) to understand the correlation between the femoral head centre and native anteversion.

## Methods

### Study participants

**Figure 1:**
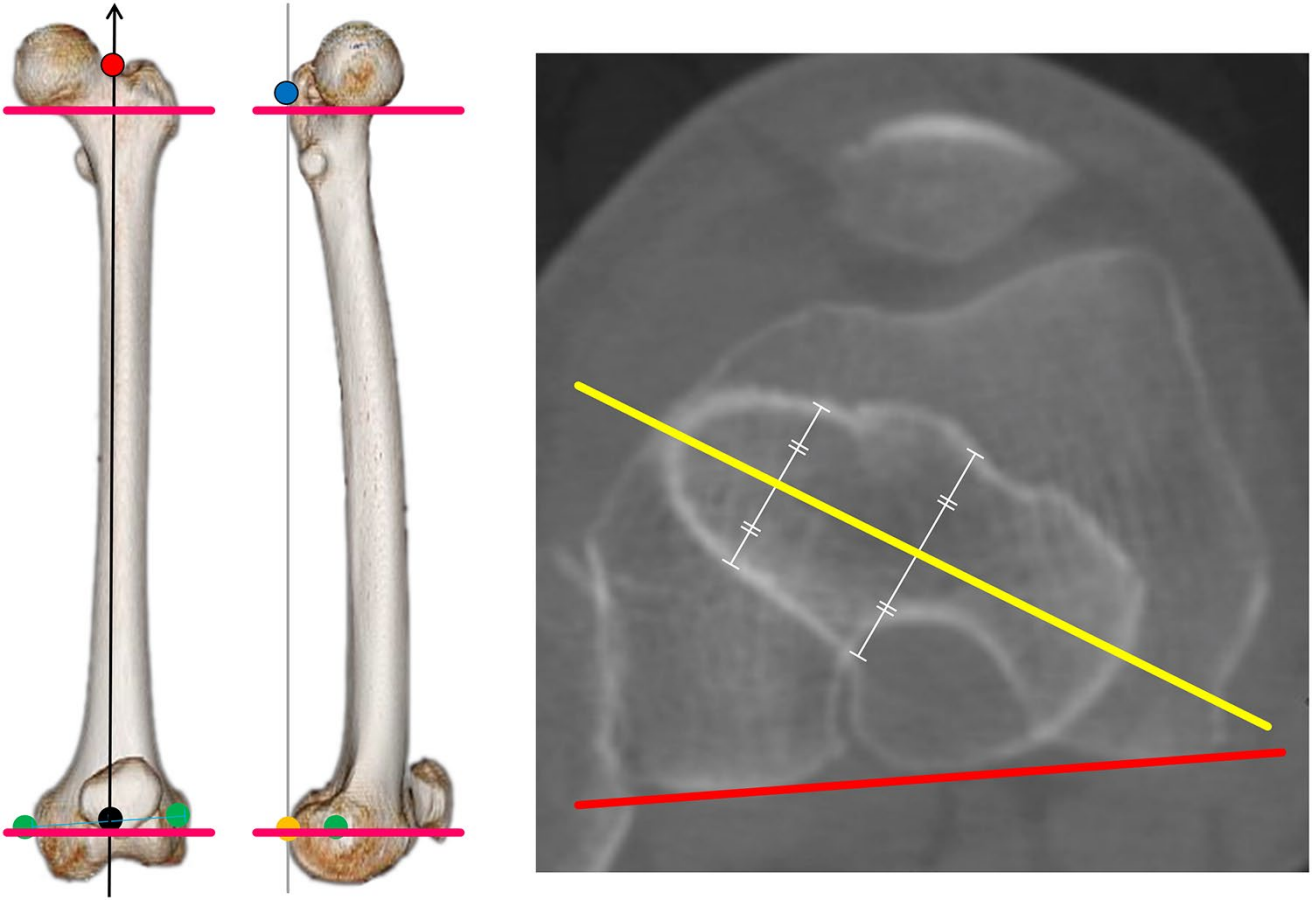
Native anteversion was determined using the method reported by Sugano et al.^9^. The coordinate system for the femur was defined relative to the posterior condylar plane of the femur, which was formed by the proximal posterior surface (blue point), lateral condyle, and medial condyle (yellow point)^10^. The z-axis was defined as a projected line onto the posterior condylar plane, passing through the knee centre (black point) and trochanteric fossa (red point). This method used the axial plane at the level just below the femoral head on the coronal plane to determine the neck axis without head centre. The angle between the neck axis (yellow line) and the posterior femoral condylar line (red line) was measured^9^.

The research protocol was approved by the Medical Ethics Committee of the Kanazawa University Advanced Science Research Center (Approval Number: 1751). All methods were carried out in accordance with the relevant guidelines and regulations. Informed consent was obtained in the form of opt-out on the web-site for this study. All methods were carried out in accordance with the relevant guidelines and regulations. This in silico retrospective study included 359 patients who had undergone primary THA at our hospital between January 2012 and March 2016 (Level of evidence: Level III). Patients with bilateral osteoarthritis and those with unclear images were excluded based on preoperative CT data. We chose patients with a normal hip and available preoperative CT data who were undergoing unilateral THA for contralateral hip disease at our institution. Normal hips were defined as those with normal morphology on radiography without osteoarthritic changes. Other exclusion criteria were the presence of pain, functional disorders, or history of hip disorder. We excluded patients with bilateral hip osteoarthritis, osteonecrosis, and rheumatoid arthritis (n = 191), hip osteotomy (n = 23), hip fracture or other disease (poliomyelitis, Perthes deformity, infection, and tumour; n = 12), hip pain (n = 9), and no preoperative CT data (n = 24). Finally, a total of 100 patients were included in this study. The patients were classified into the following 4 groups according to amount of native anteversion: less than 10°, 10–20°, 20–30°, and more than 30°. The details of patient demographics are shown in Supplementary Table 1. The amount of native anteversion was determined using the method reported by Sugano et al.^9^ (Fig. 1). This method uses the axial plane at the level just below the femoral head on the coronal plane to determine the neck axis without head centre. The angle between the neck axis and posterior femoral condylar line was measured. The coordinate system for the femur was defined relative to the posterior condylar plane of the femur, which was formed by the proximal posterior surface, lateral condyle, and medial condyle^10^.

### CT scanning and three-dimensional (3D) reconstruction

A preoperative CT scan from the iliac wing to the femoral condyle was obtained using a helical CT scanner (Lightspeed VCT; GE Medical Systems, Milwaukee, WI, USA). The slice thickness was 1 mm, and the pitch was 2.5 mm (160–250 slices depending on body size). All CT slices were saved in the Digital Imaging and Communications in Medicine (DICOM) format and imported into the CT-based templating software (ZedHip; Lexi Co., Tokyo, Japan), which was used to create virtual 3D bone models and perform simulations using the preoperative THA planning mode^11,12^.

### Definition of the coordinate system and measurement of femoral anteversion

**Figure 2:**
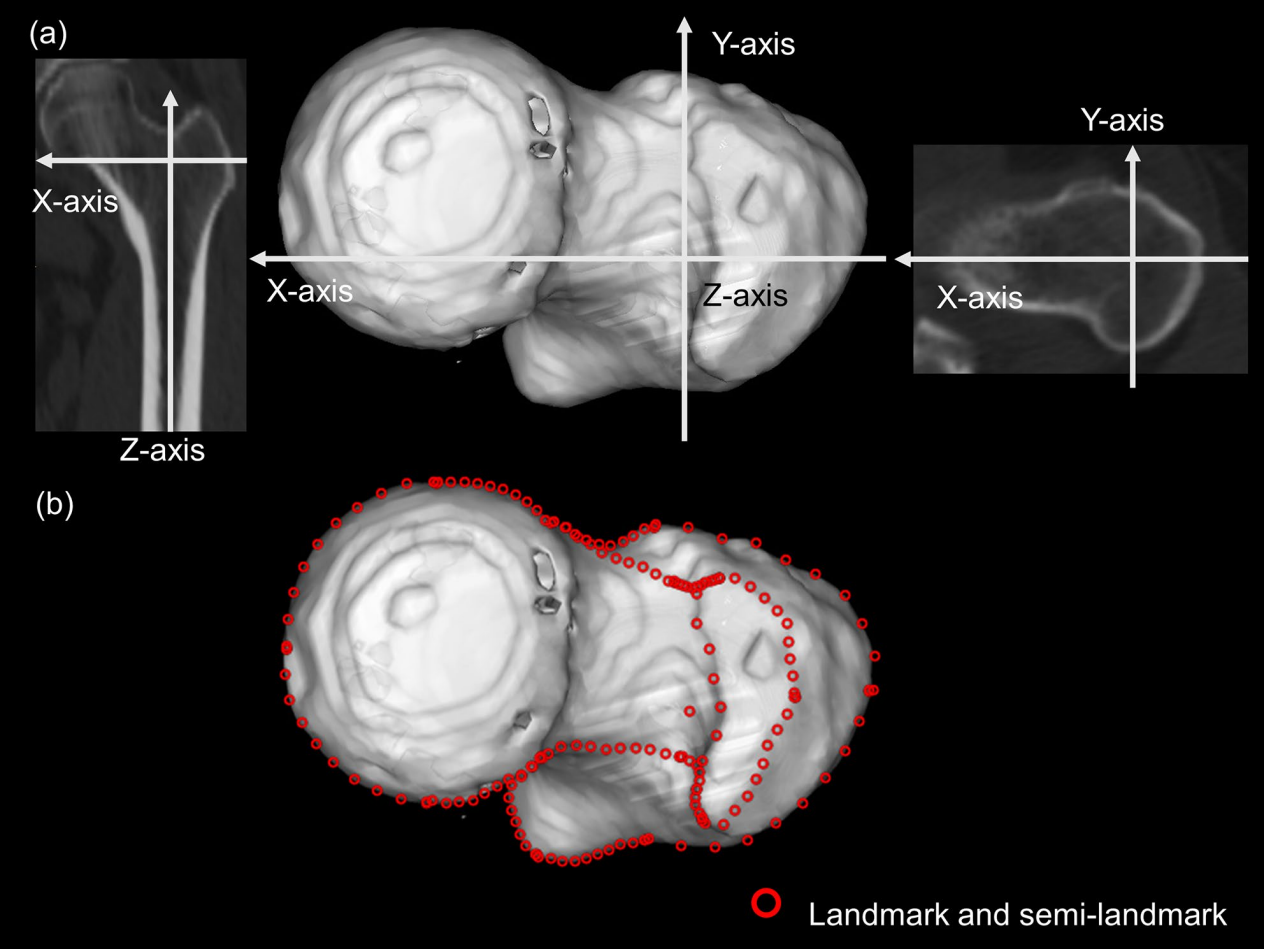
(**a**) The coordinate system of the proximal femur, in which the z-axis was defined as the line through the centre of the proximal femoral canal, the x-axis as the line parallel to the native anteversion through the z-axis, and the y-axis was perpendicular to the x- and z-axes. (**b**) Using this coordinate system, the morphology of the superior aspect of the proximal femur was analysed. Landmarks were placed on the inflection point of the femur; the detailed landmark positions are shown in Table 1. One hundred fifty semi-landmarks on the femur were manually placed and then automatically repositioned as equidistant points.

To assess the morphology of the proximal femur, we defined the coordinate system of the proximal femur, in which the z-axis was defined as the line through the centre of the proximal femoral canal, x-axis was defined as the line parallel to native anteversion through the z-axis, and y-axis was defined as a line perpendicular to the x- and z-axes (Fig. 2a).The proximal femoral bone axis (z-axis) was defined as the line between the centre of the canal at the lesser trochanter and centre of the canal at the isthmus^11^. The centre of the canal was determined by fitting the circle to the canal. During morphological analysis, the image of the 3D bone model captured from the superior vantage point was used (Fig. 2a).

### Positioning landmarks and semi-landmarks

All left femurs were flipped horizontally, so that all femurs could be analysed as if they were the right side. Then, the enlargement ratios of all images were unified using Adobe Photoshop CC 2018 (Adobe Systems Incorporated, San Jose, California, USA). Using these images, the morphology of the proximal femur was analysed using the method reported previously^12^. Landmarks were placed on the inflection point of the femur manually; the detailed landmark positions are shown in Table 1 (Fig. 2b). One hundred and fifty semi-landmarks on the femur were manually placed and then automatically repositioned as equidistant points (Fig. 2b). Ten semi-landmarks were placed between each landmark (Fig. 2b). To position the landmarks and semi-landmarks, tpsDig2 software version 2.31 was used; 22 tpsUtil32 software version 1.76 was used to create and manage the TPS file, which was designed for holding the 2D and 3D data. The TPS series, which was freely published by Rohlf et al*.*, allows statistical analysis of the landmark morphometric data by simplifying data collection and maintaining landmark data from digitised images^13^.

**Table 1:** Details of landmarks and semi-landmarks.

The number of landmarks		Semi-landmarks	
1	The most medial point of femoral head	1–2	10
2	The most posterior point of femoral head	2–3	10
3	The transition section from femoral head to neck at the posterior	3–4	10
4	Trochanteric fossa	4–5	10
5	The most posterior point of greater trochanter	5–6	10
6	The lateral point of greater trochanter	6–7	10
7	The most anterior point of greater trochanter	7–8	10
8	Anterior point of femoral neck isthmus	9–10	10
9	The transition point from femoral neck to head at anterior	10–1	10
10	The most anterior point of femoral head	5–7	10
11	The extension line of the centre of femoral canal (z-axis)	3–12	10
12	The most prominent point of lesser trochanter	12–13	10
13	The inflection point from lesser to greater trochanter	13–14	10
14	The most lateral point of greater trochanter	14–15	10
15	The anterior prominent point of greater trochanter	15–9	10

### Morphometric geometrical analysis

**Figure 3:**
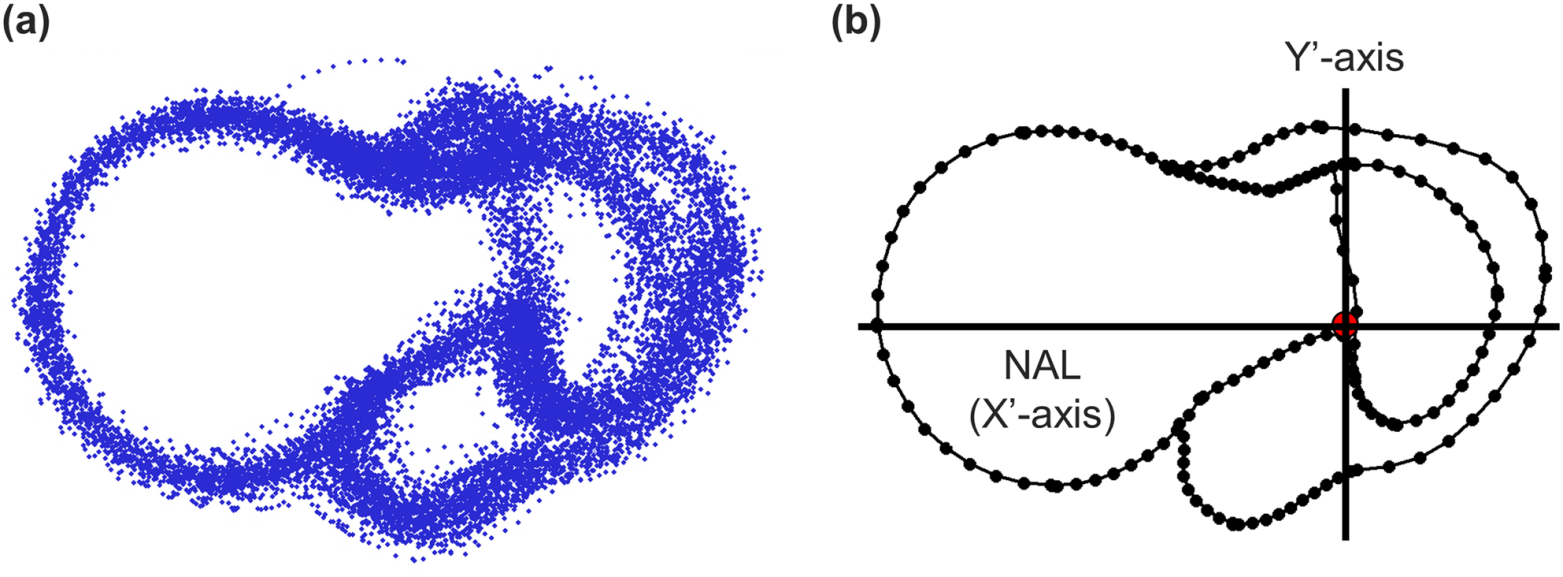
(**a**) The consensus of all landmarks using generalised Procrustes analysis was shown. (**b**) To describe major trends in shape variations within the sample, we performed a principal component analysis of the partial warp variables. The X′-axis was parallel to the native anteversion through the extension point of the femoral canal centre, which was equal to the native anteversion line (NAL). The Y′-axis was perpendicular to the X′-axis. The red point shows landmark 11, the extension line of the centre of the femoral canal (Z-axis).

The generalised orthogonal least-squares Procrustes average configuration of the landmarks was computed using the generalised Procrustes analysis (GPA) superimposition method^12–14^. GPA was performed using tpsRelw32 software version 1.69^13^. In geometrical morphometrics, the shape is defined as the information remaining after the effects of the position, orientation, and scale have been kept constant^15^. In this study, GPA was used to remove these effects in landmark and semi-landmark configurations, and the centroid size was used for size measurements. Semi-landmarks were constrained to slide along an estimated tangent at each sliding point and positioned to minimise the bending energy required for deformation of the consensus of the selected specimen following an optimisation protocol^16^. Minimising bending energy is the optimal solution for producing transformation grids between specimens^16^. The consensus of all landmarks is shown in Fig. 3a. To describe major trends in shape variations within the sample, we performed a principal component analysis of partial warp variables (relative warp analysis)^17^. The relative warps were principal component vectors in this space and were used to describe major trends in shape variations among specimens with a sample and deformation in shape^17^. The alpha parameter, which determines the relative weight of the principal warps on different scales, was fixed at 0. The shape of the femur was visually analysed using the results acquired from the relative warp analysis (Fig. 3b). In this study, the first two relative warp scores were analysed to assess the proximal femoral morphology. The coordinate system was defined as follows: the X′-axis was parallel to the native anteversion through the extension point of the femoral canal centre, and the Y′-axis was perpendicular to the X′-axis (Fig. 3b). The typical shapes of the proximal femur were created in all groups according to the amount of native anteversion.

### Measurement analysis using CT data and assessment of the correlation between the amount of native anteversion and parameters

**Figure 4:**
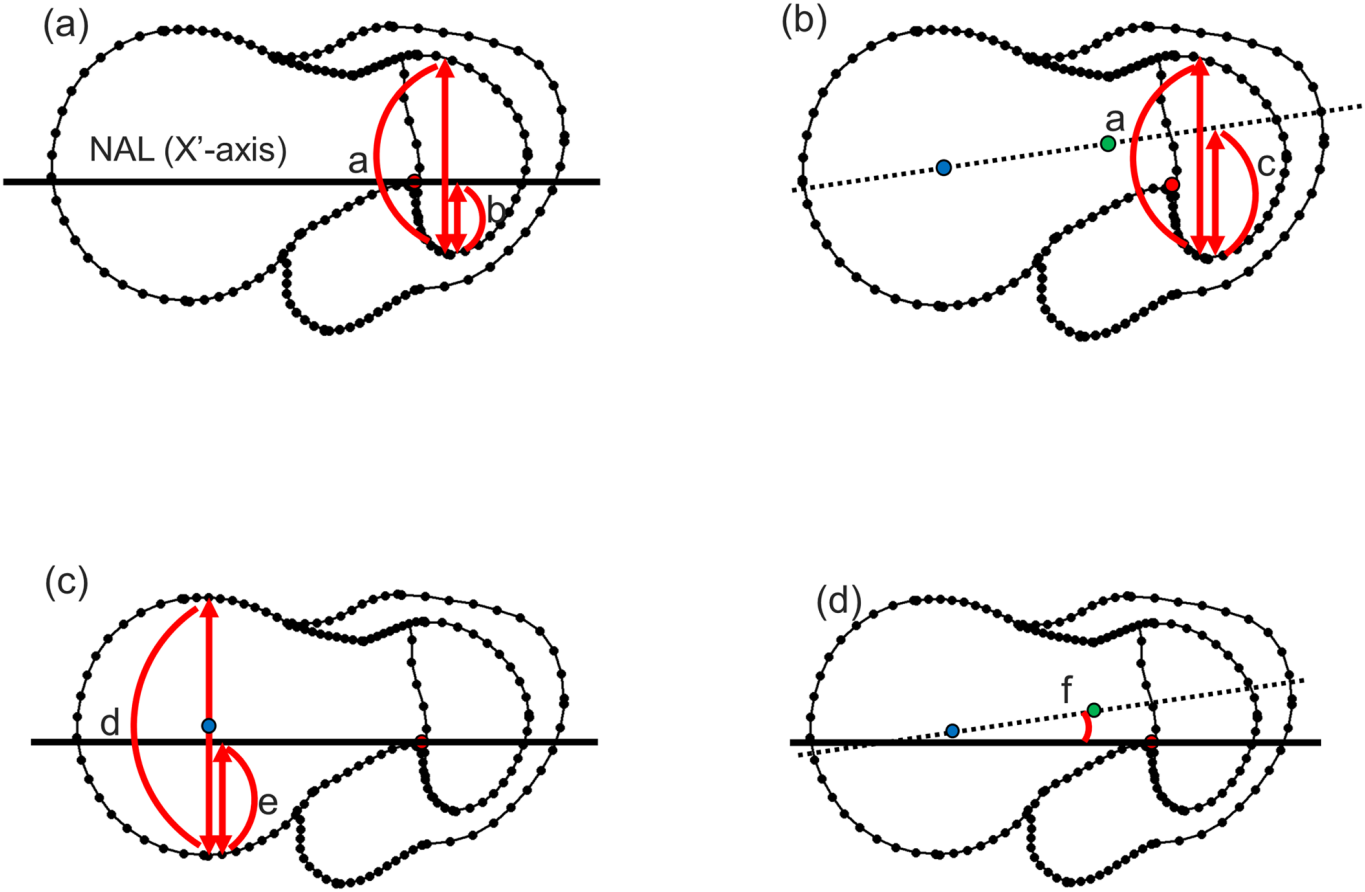
(**a**) Insertion point to the canal at the greater trochanter: NIP. NAL shows the native anteversion line (NAL), which passes the z-axis; the red point, the extension line of the centre of the femoral canal (Z-axis); a, the length from the posterior to the anterior edge of the greater trochanter; b, the length from the posterior edge to the passing point of the NAL. (**b**) Intersection point of the head-neck axis and greater trochanter: LSR. The blue point shows the femoral head centre; the green point, the neck centre at the narrowest point of the neck; c, the length from the posterior edge to the passing point of the line through the femoral head centre and the femoral neck centre (dotted line, line of head-neck) in this shape at the greater trochanter. (**c**) Passing point of the native anteversion at the femoral head: MDL. d shows the length of the femoral head diameter; and e, the length from the posteriormost point to the passing point of the NAL. (**d**) Angle between the native anteversion and head-neck axis: MAL. F shows the angle between the line of the head-neck centre (dotted line) and the native anteversion (solid line).

We assessed the following 4 parameters: the insertion point of the femoral nail to proximal femoral canal at the greater trochanter (Insertion point to canal at the greater trochanter; nail insertion point [NIP]), the intersection point of the head-neck axis and greater trochanter (intersection point of head-neck axis and greater trochanter; lag screw route [LSR]), the correlation between the line from the proximal femoral canal parallel to the native anteversion and the location of the femoral head centre (passing point of the native anteversion at the femoral head; mismatch distance of lag screw route [MDL]), and the angle between the native anteversion and head-neck axis (angle between the native anteversion and head-neck axis; mismatch angle of lag screw route [MAL]) based our hypothesis. The 4 parameters were assessed by typical shapes of the proximal femur and measured using CT data. The NIP is the passing point of the native anteversion line (NAL) that passed the z-axis at the greater trochanter (Fig. 4a). We defined (a) as the line between the most anterior point of the greater trochanter and most posterior point of the greater trochanter, which was perpendicular to the x-axis. We defined (b) as the line between the x-axis and the most posterior point of the greater trochanter, which was perpendicular to the x-axis. The LSR is the passing point of the line through the femoral head centre and femoral neck centre at the narrowest portion of the neck (line of head-neck centre) in this shape at the greater trochanter (Fig. 4b). The MDL is the passing point of the NAL at the femoral head (Fig. 4c). The MAL is the angle between the line of the head-neck centre and the native anteversion (Fig. 4d). The NIP, LSR, and MDL were expressed as percentages (calculated as b/a × 100, c/a × 100, and e/d × 100, respectively). The MAL was expressed as an angle (Fig. 4; the angle is denoted as “f”). We assessed the correlations between native anteversion and these 4 parameters using both morphometric geometric analysis and CT data. We performed a reproducibility test by doing intra- and interobserver analyses. For the intra-observer analysis, the same operator repeated the measurements of the first 20 cases, with measurements completed more than a half year apart.

### Statistical analysis

The Pearson correlation coefficient was used to measure correlations among variables. Comparisons between 4 groups were completed using ANOVA, followed by Tukey multiple means post-test. Statistical analyses were performed using Prism8 software (GraphPad, La Jolla, CA, USA). For all analyses, statistical significance was determined as *p* < 0.05.

## Results

To assess the interobserver variability, 10 cases were randomly selected and measured by a second operator (Y.Y.). In every variable, the intraclass correlation coefficient was higher than 0.8. Reproducibility and reliability results were shown in Supplementary Table 2.

**Figure 5:**
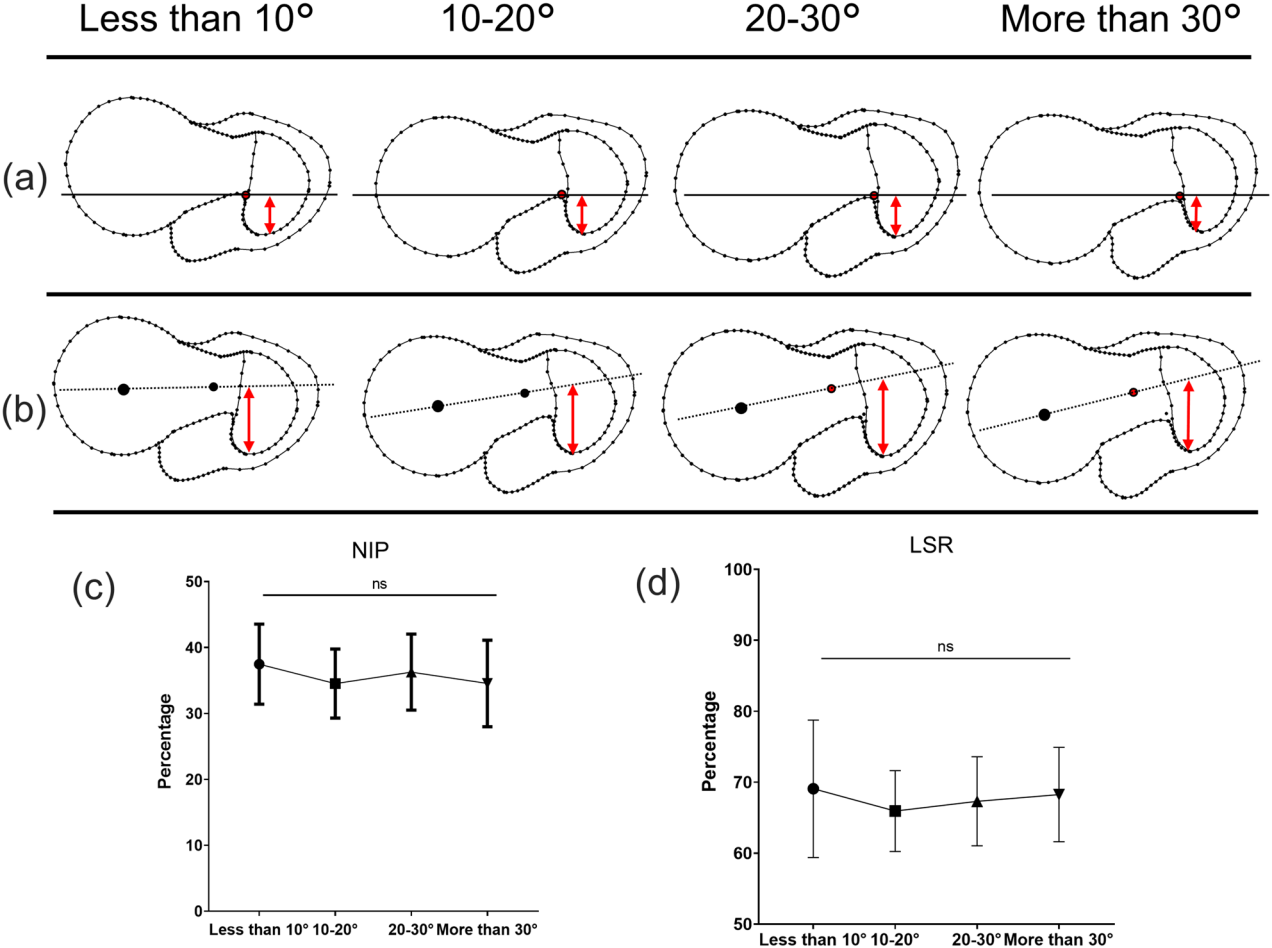
The shapes of the proximal femur according to the native anteversion, where (**a**) is the insertion point to the canal at the greater trochanter (NIP), and (**b**) is the intersection point of the head-neck axis and greater trochanter (LSR). (**c**) and (**d**) show the details and ANOVA results of NIP and LSR, respectively.

**Figure 6:**
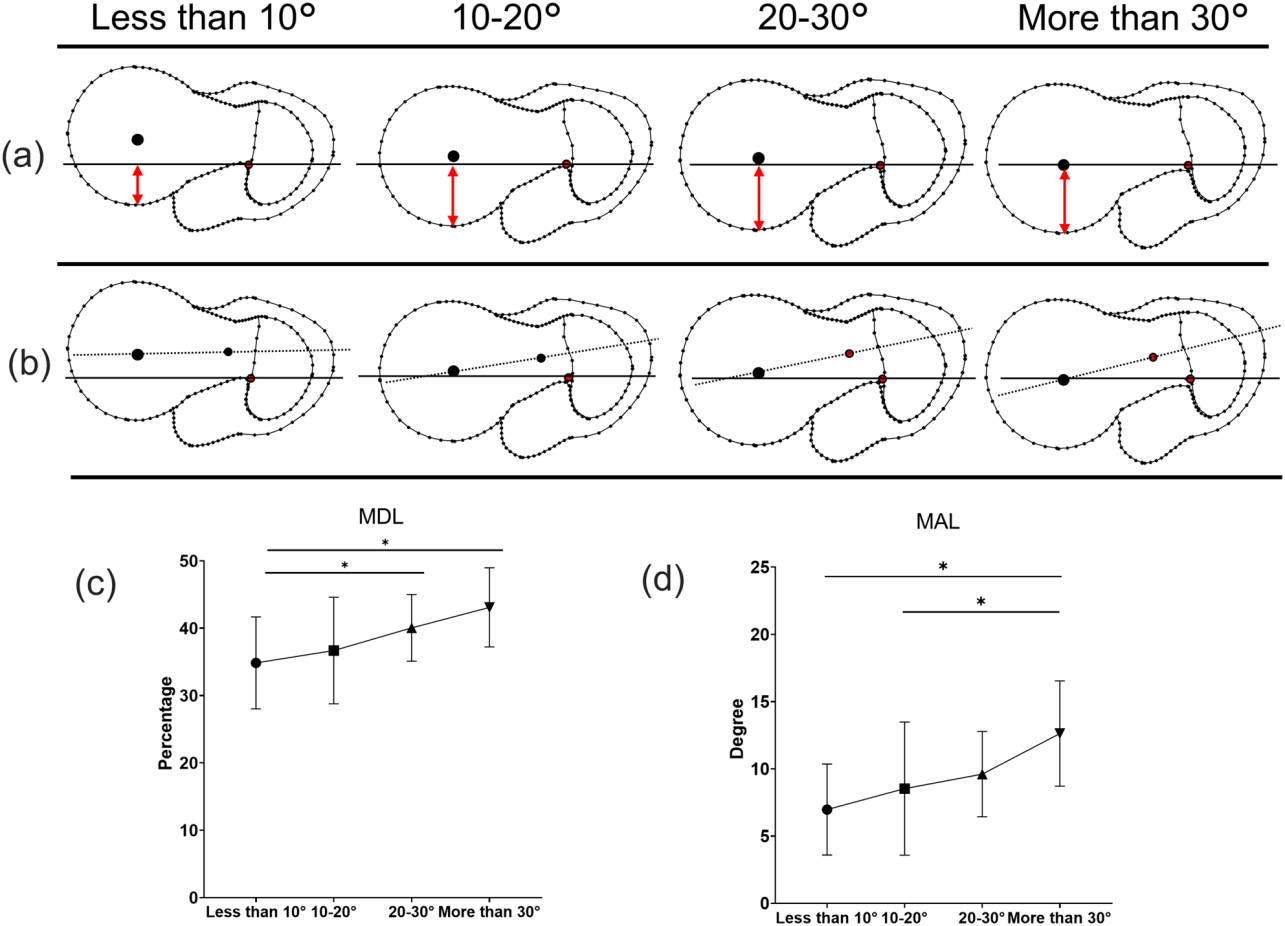
The shapes of the proximal femur according to the native anteversion, where (**a**) is the passing point of the native anteversion at the femoral head (MDL), and (**b**) is the angle between the native anteversion and head-neck axis (MAL). (**c**) and (**d**) show the details and ANOVA results of MDL and MAL, respectively.

**Figure 7:**
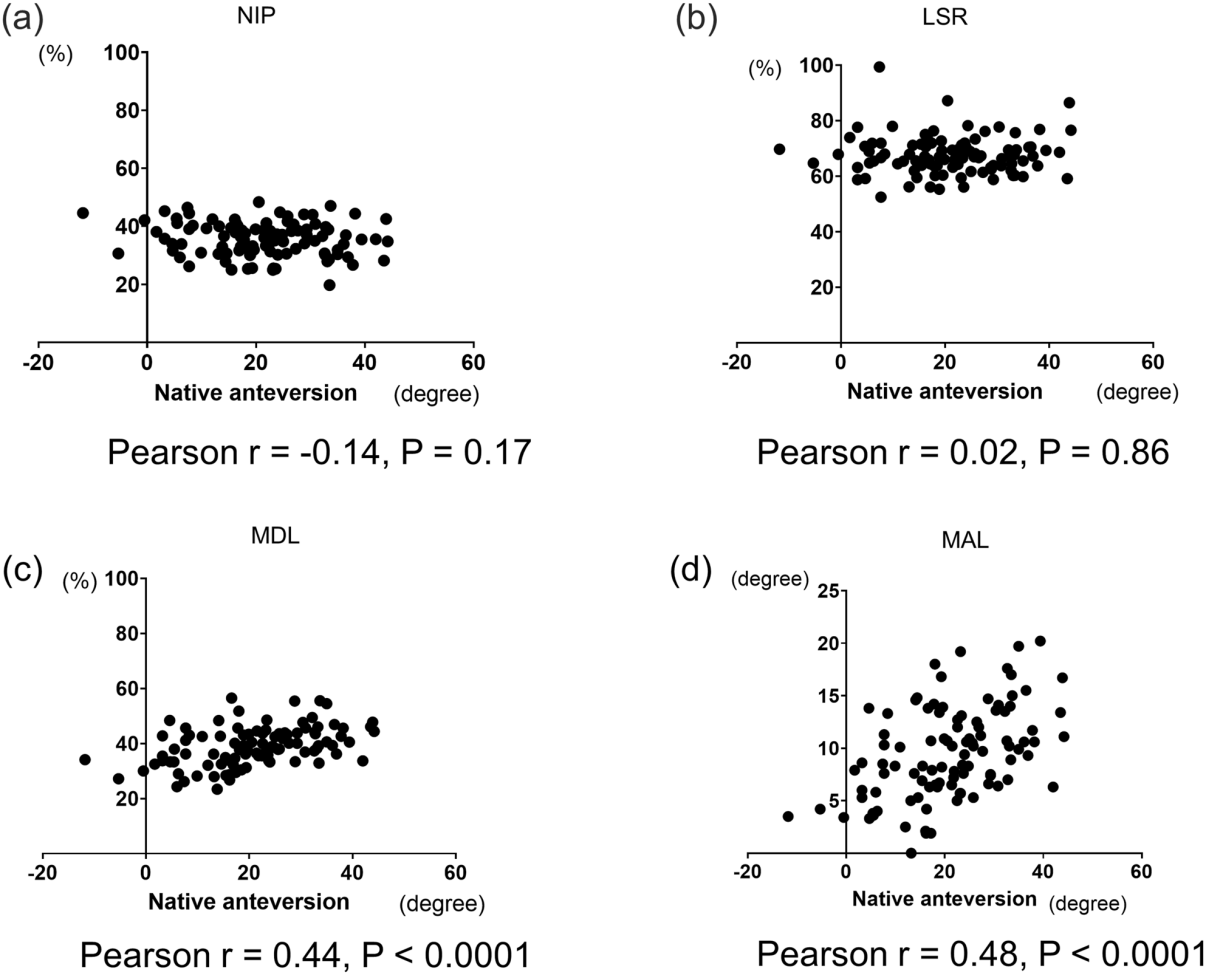
The correlations between 4 parameters and native anteversion. In all graphs, the X-axis is the native anteversion (degree). In (**a**–**c**), the Y-axis shows percentages, and, in (**d**), the Y-axis shows degrees. The insertion point to the canal at the greater trochanter, the intersection point of the head-neck axis and greater trochanter, and the passing point of the native anteversion at the femoral head were expressed as percentages (calculated as b/a × 100, c/a × 100, and e/d × 100, respectively).

The shapes of the proximal femur according to the amount of native anteversion and the measurement of the parameters using CT data are shown in Figs. 5 and 6. In the NIP, the passing point of the NAL was located more posteriorly (mean 35.6%) at the greater trochanter (Fig. 5a and c), although the line of the head-neck centre passed more anteriorly at the greater trochanter (mean 67.5%) in the LSR (Fig. 5b and d). There was no significant difference among 4 groups according to native anteversion in the NIP and LSR (Fig. 5c and d). The NIP and LSR were not correlated with native anteversion. Especially, the LSR was almost the same percentage regardless of the amount of native anteversion (Fig. 7a and b). In the MDL, NAL passed posterior to the femoral neck and head (Fig. 6a and c), especially in the group with less than 10° native anteversion. ANOVA showed a significant difference among groups according to the amount of native anteversion in the MDL and MAL (Fig. 6c and d). The MDL was significantly correlated with the amount of native anteversion (Fig. 7c). The MAL showed the difference in this angle according to amount of native anteversion, wherein the angle decreased in the group with less than 10° but increased in the group with more than 30° (Fig. 6b and d). There was a positive correlation between the MAL and amount of native anteversion (Fig. 7d).

## Discussion

This study confirmed a correlation between the characteristics of the morphology of the proximal femur superior aspect and amount of native anteversion. Morphometric geometric analysis showed that the proximal femoral canal and line of the head-neck centre were skew lines. The clinical significance of this correlation is that if the short femoral nail is inserted into the femoral canal centre, the lag screw parallel to the native anteversion is routed posteriorly at the femoral neck and head. The MDL showed that this mismatch increased as the native anteversion decreased.

The correct introduction of an intramedullary nail in the treatment of fractures of the long bone prevents malrotation, iatrogenic fracture, gapping, and hoop stresses^5,18^. A previous cadaveric study recommended that the entry point of intramedullary nail be 5 mm posterior to the apparent apex of the greater trochanter^5^. Our results exhibiting the entry point to the femoral canal at 35.6% posterior at the greater trochanter agreed with the results of this previous study. However, this entry point was not considered with the route of the lag screw. Another previous biomechanical analysis suggested that central placement of the lag screw on lateral radiography was recommended^19^. If the route of the lag screw was intended to be positioned in the centre of the neck and head of the femur, the entry point of the short femoral nail translated anteriorly, which was shown by parameter 2 in the present study. This anterior insertion point was constant in our analysis, which was a mean 67.5% anterior from the posterior of the greater trochanter. However, from this insertion point, the femoral nail was inserted from anterior to posterior in the sagittal plane, which is problematic. Therefore, in patients with a narrow canal, this anterior insertion is impossible. This mismatch could occur due to the origin of the femoral neck at the anterior portion of the femoral metaphysis. A previous CT-based study suggested that the proximal femoral metaphysis presented a highly variable anterior flare and torsion^20^. This finding suggests that the femoral neck started at the anterior portion of the femoral metaphysis. Therefore, our findings showed that the entry point to the femoral canal was posterior, but, from this entry point, the lag screw could not be positioned in the centre in the anteroposterior plane. If surgeons desire to position the lag screw in the centre of the femoral neck in the anteroposterior plane, the entry point of the femoral nail should be translated as anteriorly as possible.

Our findings could be applied to THA. Several studies have previously investigated proximal femoral markers for native femoral version related to implant version in THA^6^. Prior studies have found a fixed relationship between the lesser trochanteric line and femoral neck axis as well as that with the transverse axis of the lesser trochanter and femoral posterior condylar axis^21,22^. Furthermore, a previous study suggested that it was possible to determine femoral version based solely on the proximal femoral anatomy without the additional expense and radiation required to image the distal femur^6^. They reported that the angle between the maximum canal diameter centred on the greater trochanter and femoral neck axis had relatively low variability^6^. Our data contributed novel information to the realm of orthopaedics, in that the angle between the line of the head-neck centre and native anteversion showed a weak correlation. The difference between these 2 axes indicates that the neck axis at the height of the lowest point of the femoral head and neck axis at the height of the femoral head was different, showing that the femoral neck had torsion. This torsion was reported previously^9,20,23^. Torsion indicates the difference of the angle of the axis between the different two slices in the axial plane of the femur, while anteversion was defined as the angle between the neck axis and table top plane; as reported previously^20,23^. A previous morphologic study reported that the increased torsion of the hip femora was exhibited within the diaphysis between the lesser trochanter and isthmus in patients with developmental hip dysplasia^9^. We found that the femoral neck itself had torsion and that this torsion might be correlated with the amount of native anteversion. These findings were important to reconstruct the anatomical head centre in THA. The lateral femoral offset was reported to be correlated with abductor function and increased wear^24,25^. However, few reports assessed anterior femoral offset^26,27^. The reconstruction of the anterior anatomical head centre of the femur is important to achieve sufficient range of motion^26^. Therefore, the reconstruction of the anterior anatomical hip centre is necessary in THA. Therefore, more anteversion might be necessary to achieve an anterior femoral head centre, especially in patients with native anteversion less than 10° (Fig. 6). However, the correlation was not high (Pearson r = 0.48); thus, the MAL itself could not be a predictor of native anteversion.

This study had several limitations. First, there is user bias in the selection of landmarks. To help eliminate different biases in terms of selection of corrected points, one user was responsible for all measurements, and a zoom feature of the software was utilised. Second, we only included Asian patients, and thus, the results may not be generalisable to other populations. Furthermore, this study did not consider differences between sex. Male patients were relatively few in number. This was due to the patient selection protocol. This study included patients with no arthritic changes on the contralateral side who had undergone THA in our hospital. Therefore, the female patients who had osteoarthritis due to developmental dysplasia of the hip comprised the majority of the patients included in this study. Thirdly, this study used the coordinate system, wherein the x-axis was the NAL, because our study’s purpose was to assess the correlation between native anteversion and morphology of the superior aspect of the femur. However, the lag screw must go into the femoral head centre. Thus, there was discrepancy between our results and the clinical interpretation.

In summary, morphometric geometric analysis showed that a line from the femoral head to neck passed the anterior aspect of the greater trochanter and did not pass the femoral canal posterior to the greater trochanter. Therefore, from the insertion point of the femoral nail to the femoral canal, the lag screw according to native anteversion passed posterior to the neck and head. This mismatch was associated with native anteversion. For the central placement of the lag screw, anterior insertion of the femoral nail was needed. This finding could also be applied to THA to reproduce the femoral head centre, and more anteversion was needed than native anteversion especially in the low anteverted femur.

## Supplementary Information

Supplementary Information 1.

Supplementary Information 2.

## Data Availability

All the data used to draw the conclusions of this paper are available in the data presented in the figures and/or tables. The raw/processed data required to reproduce these findings are available from the corresponding author upon request.
