# Supplementary material for: Correlation between lag screw route and the ideal insertion point of the intramedullary nail
Source: Sci Rep. 2021 Jul 2;11:13750. doi: 10.1038/s41598-021-93348-9 (PMC8253735; doi:10.1038/s41598-021-93348-9)
Supplement: Supplementary file 1 — Supplementary Information 1. [file 41598_2021_93348_MOESM1_ESM.docx]

| Supplementary Table 1. Comparison of characteristics according to amount of native anteversion | | | | | |
| --- | --- | --- | --- | --- | --- |
|  | Total | Native anteversion | | | |
|  |  | Less than 10°,  N = 19 | 10-20°,  N = 28 | 20-30°,  N = 29 | More than 30°,  N = 24 |
| Mean anteversion (SD; range) | 21.1 (11.4; -11.8-44.2) | 3.9 (5.1; -11.8 to 9.9) | 16.4 (2.4; 10.9-19.9) | 24.7 (2.5; 20.5-29.3) | 35.7 (4.1; 30.4 to 44.2) |
| Mean age, years (SD; range) | 62.9 (10.4; 34-86) | 69.9 (7.6; 54-84) | 63.3 (9.2; 46-86) | 60.8 (10.8; 34-86) | 59.5 (10.3; 36 to 84) |
| Sex, n |  |  |  |  |  |
| Male | 19 | 5 | 5 | 6 | 3 |
| Female | 81 | 14 | 23 | 23 | 21 |
| Side, n |  |  |  |  |  |
| Right | 46 | 5 | 16 | 14 | 11 |
| Left | 54 | 14 | 12 | 15 | 13 |
| Mean height, cm (SD; range) | 155.1 (8.0; 137.1-179.0) | 156.3 (8.3; 146.0-174.0) | 153.5 (9.0; 140.0-179.0) | 156.0 (6.9; 137.1-168.5) | 155.0 (7.6; 140.0 to 173.0) |
| Mean weight, kg (SD; range) | 56.4 (10.7; 37.1-95.3)) | 60.0 (9.5; 45.0-82.7) | 54.2 (10.7; 40.3-85.0) | 58.4 (8.1; 37.1-78.0) | 53.5 (12.7; 38.5 to 95.3) |

SD, standard deviation
