# Supplementary material for: Correlation between lag screw route and the ideal insertion point of the intramedullary nail
Source: Sci Rep. 2021 Jul 2;11:13750. doi: 10.1038/s41598-021-93348-9 (PMC8253735; doi:10.1038/s41598-021-93348-9)
Supplement: Supplementary file 2 — Supplementary Information 2. [file 41598_2021_93348_MOESM2_ESM.docx]

| Table 2. Reproducibility and reliability results | | |
| --- | --- | --- |
| Analysis | Variable | ICC |
| Intra-observer (n = 20) | NIP | 0.96 |
|  | LSR | 0.8 |
|  | MDL | 0.95 |
|  | MAL | 0.88 |
| Interobserver (n = 10) | NIP | 0.94 |
|  | LSR | 0.86 |
|  | MDL | 0.92 |
|  | MAL | 0.9 |

ICC, intraclass correlation coefficients; NIP, nail insertion point; LSR, lag screw route; MDL, mismatch distance of lag screw route; MAL, mismatch angle of lag screw route.
